# 

**Published:** 1813-11

**Authors:** 


### MONTHLY CATALOGUE OF MEDICAL BOOKS.

THE Edinburgh New Dispensatory, containing the Elements of Materia Medica and Pharmacy, with accurate Translations of the London, Dublin, and Edinburgh Pharmacopœia. By John Thomson, M.D.—Highley and Son.

A Practical Materia Medica, in which the various Articles are fully described and divided into Classes and Orders, according to their effects; their Virtues, Doses, and the Diseases in which they are proper to be exhibited are fully pointed out: interspersed with practical Remarks and some select Formulæ; to which is added, a general Posological Table, intended principally for the use of Students and junior Practitioners. Second Edition. 12mo.—Highley and Son.

The Anatomical Instructor, or an Illustration of the Modern and most approved methods of Preparing and Preserving the different Parts of the Human Body, and of Quadrupeds, by Injection, Corrosion, Maceration, Distension, Articulation, Modelling, &c. with a variety of copper-plates. By Thomas Pole, Member of the Royal College of Surgeons. A new edition, with additional notes. 12mo. —Callow and Underwood.

An Essay on the Philosophy, Study, and Use of Natural History. By Charles Fothergill. 12mo.—White and Co.

The Art of Preserving the Sight unimpaired to an extreme Old Age, and of re-establishing and strengthening it when it is become weak. 12mo.—Colburn.

Some Account of an uncommon Appearance in the Flesh of a Sheep, with Reflections on the Nutrition of Sheep, &c. By W. Vaughan, M.D. 8vo.—Harding.

A brief Description of the Plague, with Observations on its Prevention and Cure. By R. Pearson, M.D. 8vo.—Underwood.

Floria Glottiana: being a Catalogue of the Indigenous Plants on the banks of the River Clyde, and in the Neighbourhood of the City of Glasgow. By Thomas Hopkirk, Fellow of the Linnæan Society, &c.

A General Account of the Hunterian Museum, Glasgow: including Historical and Scientific Notices of the various objects of Art, Literature, Natural History, Anatomical Preparations, Antiquities, &c. in that celebrated Collection. By Captain J. Laskey. 8vo.—Longman and Co.

---

### *NOTICES TO CORRESPONDENTS.*

*Communications from the following gentlemen will appear in our next Number:—Dr. Uwins; Messrs. D. H. Davies, R. Wright, Charles Mayo, W. Jones; C. W. S.; an Apothecary, &c. Dr. Jeffrey's letter will be attended to.*

*All Communications are requested to be addressed to the care of the Publisher, No. 1, Paternoster Row.*

On

### *ON THE COMPLETION OF SETS.*

*THE Series of the Medical and Physical Journal have for several years past been acknowledged to constitute the most valuable periodical collection of important facts and discoveries in Medical Practice, which exist in any language. It is now Seventeen Years since the work first claimed the notice of the Public, and during this period of unparalleled activity in Scientific and Medical Research, it has been the depository of the communications of the Faculty in every part of the world; while its editors have never failed to glean, from every authentic source, whatever appeared to them to be worthy of the attention of their readers. Hence it is to assume nothing beyond the strict fact to state, that the London Medical Journal now is, and long has been,* THE UNIVERSAL GAZETTE OF THE FACULTY, *circulating wherever the English Language is read, and that circulation reflecting, as its natural effect, a universality of respectable correspondence unequalled in the experience of Periodical Literature.*

*Under these circumstances, the* THIRTY VOLUMES, *of which the entire Series now consist, have acquired a value and importance which the Public, and even the sanguine wishes of the Proprietors, could never have anticipated. At the same time the increasing length and corresponding value of this series have been attended by a corresponding inconvenience to the friends of the work; and many Gentlemen express their regret at the loss and imperfection of Numbers and Volumes, by which their Sets are broken, while they complain of the great expence attending the purchase of the Numbers in which they are deficient, at the full, but necessary price of* HALF-A-CROWN. *On their parts the Proprietors are anxious to make such present sacrifices as may gratify their friends, and perhaps ultimately accord with the interests of the work; they have therefore resolved, during a period of* THREE MONTHS, *i. e. from the 1st of November to the 1st of February next, to vend all the Numbers published prior to the present year at* EIGHTEEN-PENCE PER NUMBER, *or Half-a-Guinea per Volume half-bound.*

*This temporary concession on their parts will, they trust, prove agreeable to many of the earliest and best friends of the work, who are also the most anxious to perfect their Series, and serve as an accommodation to the funds of Medical Reading Societies, now established in many districts, part of whose Library-Stock consists in general of parts of the Series of this work.*

*As the perfecting of Sets will naturally lead to a desire to possess a* GENERAL INDEX TO THE WHOLE, *this seems to be a suitable occasion on which to re-announce that a General Index will be put to press as soon as Five Hundred Names are received at the Office of the Publisher, implying a disposition to pay* ONE GUINEA *for the same on its delivery.*

